# Nutritional Programming via Maternal Protein Restriction Mitigates Long-Term Consequences of Acute Kidney Injury

**DOI:** 10.3390/ijms27208974

**Published:** 2026-10-10

**Authors:** Luis Enrique García-Ortuño, Karla Barragán, Rebecca Caldiño-Bhon, Emma Luna, Rosalba Pérez-Villalva, Norma A. Bobadilla

**Affiliations:** 1 Molecular Physiology Unit, Instituto de Investigaciones Biomédicas, Universidad Nacional Autónoma de México, Ciudad de México 04510, Mexico; mvz_luisenrique@yahoo.com.mx (L.E.G.-O.); karla.barragan.jimenez@gmail.com (K.B.); caldino.rebecca@ciencias.unam.mx (R.C.-B.); emma@ciencias.unam.mx (E.L.); melibiosa@hotmail.com (R.P.-V.); 2 Department of Nephrology and Mineral Metabolism, Instituto Nacional de Ciencias Médicas y Nutrición Salvador Zubirán, Ciudad de México 14080, Mexico; 3 Facultad de Medicina Veterinaria y Zootecnia, Universidad Nacional Autónoma de México, Ciudad de México 04510, Mexico

**Keywords:** renal fibrosis, acute kidney injury to chronic kidney disease transition, oxidative stress, renal resilience

## Abstract

The kidney is highly sensitive to prenatal and early-life environmental influences. Maternal protein restriction (MPR) can alter renal development and may modify susceptibility to chronic kidney disease (CKD) following acute kidney injury (AKI). We investigated whether MPR during gestation and/or lactation influences the progression from AKI to CKD after renal ischemia–reperfusion (IR) in rat offspring. Fifty Wistar pregnant rats were fed a control (20% casein) or protein-restricted (10% casein) diet during gestation, with diets maintained or switched during lactation, generating four maternal nutritional groups (CC, CR, RC, and RR). At 280–330 g body weight, offspring from these groups were randomly assigned to left renal IR plus right nephrectomy (CC + IR, CR + IR, RC + IR, and RR + IR) or right nephrectomy alone (CC, CR, RC, and RR). Renal outcomes were assessed in two independent cohorts of rats at 7 days and 5 months after surgery. Offspring from MPR during gestation and/or lactation (CR + IR, RC + IR, and RR + IR) showed reduced susceptibility to AKI-to-CKD progression compared to the CC + IR group, characterized by attenuated proteinuria, absence of glomerular hypertrophy, and minimal renal fibrosis. This protective phenotype was associated with reduced oxidative stress and endoplasmic reticulum stress, as indicated by lower 4-HNE, CHOP, and BiP, together with enhanced post-ischemic repair reflected by lower urinary KIM-1 and Hsp72 excretion. MPR during gestation and/or lactation induces a persistent renal phenotype in the offspring that confers resistance to AKI-to-CKD progression, potentially through modulation of oxidative and endoplasmic reticulum stress. These findings highlight developmental nutritional programming as a previously underappreciated determinant of renal resilience in offspring after AKI and provide a basis for investigating nutritional and developmental mechanisms that may be targeted to prevent CKD after AKI.

## 1. Introduction

Exposure to adverse events during prenatal and early postnatal development can induce persistent changes in organ structure, physiology, metabolism, and gene regulation, thereby influencing disease susceptibility later in life [1,2,3,4]. The kidney is particularly vulnerable to developmental insults because nephrogenesis occurs within a defined developmental window and can be permanently influenced by intrauterine and early-life conditions. Maternal undernutrition, intrauterine growth restriction, prematurity, pharmacological exposure, and environmental contaminants have been associated with impaired renal development and reduced nephron endowment, contributing to substantial interindividual variation in nephron number at birth [5,6,7]. Recent experimental evidence further indicates that maternal nutritional status can influence nephron progenitor cell differentiation, branching morphogenesis, and the molecular programs governing nephron formation [8].

MPR is one of the most widely used experimental models to investigate the developmental programming of renal structure and function. Protein restriction during gestation reduces nephron endowment by approximately 20–30%, depending on the severity and timing of the dietary intervention, and may induce compensatory glomerular hypertrophy, altered renal hemodynamics, and hypertension in the offspring [9,10,11]. Reduced nephron endowment is clinically relevant because it has been associated with increased susceptibility to hypertension and chronic kidney disease (CKD) later in life [10,12,13].

However, developmental responses to an adverse environment are not necessarily exclusively detrimental. Developmental plasticity allows the developing organism to modify its structural, physiological, and metabolic phenotype in response to environmental conditions [14]. From an evolutionary perspective, such adaptations may enhance survival under adverse conditions, although they may also impose long-term costs [15]. Consequently, the effects of maternal nutritional restriction may depend not only on its impact on renal development but also on how the programmed kidney responds to subsequent physiological or pathological challenges.

Consistent with this concept, our previous studies demonstrated an unexpected adaptive phenotype in offspring exposed to moderate MPR. Despite a reduction in nephron number, offspring subjected to a 50% reduction in maternal dietary protein during pregnancy, lactation, or both exhibited greater renal resilience following 24 h of bilateral renal ischemia/reperfusion (IR). This response was characterized by efficient recovery of renal function, improved renal blood flow (RBF), enhanced antioxidant defenses, and activation of hypoxia-inducible factor-1α (HIF-1α) signaling [16]. These findings indicate that developmental nutritional programming can modify the response of the mature kidney to an acute injury. However, whether this apparent early-life resilience persists after acute kidney injury (AKI) and influences long-term renal outcomes remains unknown.

AKI is increasingly recognized as an important risk factor for subsequent CKD rather than an isolated and fully reversible event [17,18,19]. Recovery from AKI depends on the balance between adaptive and maladaptive repair. Adaptive repair involves restoration of tubular epithelial integrity, resolution of inflammation, and recovery of renal function, whereas maladaptive repair is associated with persistent tubular injury, inflammatory activation, cellular stress, metabolic and mitochondrial dysfunction, cellular senescence or cell-cycle arrest, microvascular alterations, and progressive extracellular matrix accumulation. These processes can ultimately lead to tubulointerstitial fibrosis, a defining feature of CKD. In particular, incomplete tubular repair and persistent epithelial stress can promote the production of profibrotic and proinflammatory mediators, including transforming growth factor-β (TGF-β), which contributes to immune-cell recruitment, fibroblast activation, and deposition of extracellular matrix proteins such as α-smooth muscle actin (α-SMA), fibronectin, and collagen [20,21,22,23].

Despite substantial progress in understanding the mechanisms underlying AKI-to-CKD progression, several important knowledge gaps remain. First, it is unclear whether developmental nutritional programming modifies the long-term trajectory of AKI beyond its effects on the initial acute response. Second, although MPR reduces nephron endowment, it remains unknown whether this programmed renal phenotype increases susceptibility to maladaptive repair or, conversely, confers persistent resilience following renal injury. Noteworthy, current models of AKI-to-CKD progression have largely focused on characteristics of the acute insult and renal injury itself; however, the contribution of pre-existing developmental determinants of renal resilience remains poorly characterized.

To address these unresolved questions, we investigated whether MPR during gestation and/or lactation modifies the short- and long-term renal response to IR injury in adult offspring. Using four maternal nutritional paradigms—control throughout gestation and lactation (CC), control during gestation followed by protein restriction during lactation (CR), protein restriction during gestation followed by control during lactation (RC), and protein restriction throughout both periods (RR)—we subsequently subjected adult offspring to left renal IR combined with right nephrectomy or to right nephrectomy alone. Renal outcomes were assessed at 7 days and 5 months after surgery, allowing us to distinguish acute responses from long-term consequences and to determine whether developmental nutritional exposure during gestation and/or lactation influences the progression from AKI to CKD. We further evaluated renal injury and repair, proteinuria, glomerular and tubulointerstitial remodeling, fibrosis, oxidative stress, and endoplasmic reticulum stress. This approach was designed to determine whether the previously observed early renal resilience associated with MPR represents a transient adaptive response or a persistent developmental phenotype that protects the offspring against AKI-to-CKD progression.

## 2. Results

### 2.1. Maternal Body Weight (BW) During Gestation and Lactation

Table 1 summarizes the mean maternal BW of dams fed a CC, RC, CR, or RR diet. During the gestation period, all pregnant rats showed a significant increase in BW from day 15 onward; however, in the CC and CR groups, this increase was already evident by day 10. By the end of the gestation, BW was comparable across all groups as expected, given that all diets were isocaloric. Following parturition, maternal BW decreased, returning to pre-gestational values and remaining stable throughout lactation, with no differences observed among the four groups.

### 2.2. Postnatal BW in Offspring Exposed to Different Maternal Protein Diets

At birth, no significant differences were observed among the offspring groups in BW, body length, body width, or cranial width (Table 2 and Supplementary Figure S1). However, BW was significantly lower in the CR and RR groups during the first 30 days after birth (Table 3).

### 2.3. BW and Body Composition of Adult Offspring Following Maternal Nutritional Exposure in the Long-Term Cohort (5 Months)

Once the offspring reached 280–330 g of BW, male rats from each group underwent either sham surgery or IR combined with right nephrectomy (IR + Nx). Figure 1 shows BW and body composition of offspring from CC, CR, RC, and RR dams that underwent either sham surgery or IR. Food intake was slightly lower compared to the CC group, except for the CR + IR group. Despite this, BW was similar across all groups, although the RR and RR + IR groups showed a tendency toward lower BW compared to the CC group (*p* = 0.14 and *p* = 0.15, respectively). At the end of the study, body composition analysis revealed no significant differences in fat or lean mass percentages among the groups.

### 2.4. MPR Preserves Renal Function and Limits Long-Term AKI-to-CKD Progression

Twenty-four hours after IR + Nx, CrCl, proteinuria, and urinary H_2_O_2_ excretion were evaluated. As expected, CrCl was markedly reduced in all IR groups compared with their respective controls (Figure 2A). This reduction was accompanied by a significant increase in proteinuria in the CR + IR, RC + IR, and RR + IR groups (Figure 2B). These findings confirm the successful induction of AKI severity across groups, indicating that the initial renal dysfunction induced was comparable among the studied groups and the differences seen in the long-term outcomes were primarily due to the maternal dietary regimen of each group. Notably, urinary H_2_O_2_ excretion measured 24 h post-IR shows a significant increase only in the CC + IR group when compared with all other groups. Consistent with previous findings, MPR is associated with lesser production of reactive oxygen species (ROS) following IR injury (Figure 2C) [16].

For long-term evaluation, proteinuria, RBF, and CrCl were assessed five months after IR + Nx. As expected, and in agreement with previous reports [24,25,26], the CC + IR group showed a progressive increase in proteinuria, with statistically significant differences detected as early as two months post-IR. In contrast, no significant changes in proteinuria were observed in the CR + IR and RR + IR groups throughout the study, either over time or in comparison with their respective controls (Figure 2D). These findings suggest a degree of nephroprotection in offspring born to dams subjected to protein restriction during gestation, lactation, or both periods. No significant differences were observed among the groups in BW, mean arterial pressure (MAP), RBF, or CrCl (Figure 2E–H). Kidney weight was significantly increased in the CC + IR, CR + IR, and RC + IR groups compared with their respective control groups; however, this effect was not observed in the RR + IR group, which differed significantly from the other IR + Nx groups (Figure 2I). Notably, despite greater renal damage, as indicated by progressive proteinuria, the CC + IR group maintained preserved renal function. This preservation is likely attributable to compensatory hemodynamic adaptations following nephrectomy, which sustained glomerular filtration rate (GFR) and is consistent with the glomerular diameters analysis shown in Figure 3.

### 2.5. MPR Prevents Maladaptive Glomerular Hypertrophy After Ischemic Kidney Injury

Glomerular hypertrophy was evaluated by measuring the glomerular diameters, which were categorized into seven ranges: <80, 81–100, 101–120, 121–140, 141–160, 161–180, and >180 µm. The four control groups (CC, RC, CR, and RR) subjected to nephrectomy exhibited a bell-shaped distribution of glomerular diameters, with most glomeruli falling within the 100–160 µm range (Figure 3A–D). As previously reported [27,28], the AKI-to-CKD transition in the CC + IR group was associated with glomerular hypertrophy, characterized by a significant proportion of glomeruli (approximately 40%) with diameters >160 µm (Figure 3E). Interestingly, the CR + IR, RC + IR, and RR + IR groups did not develop glomerular hypertrophy and maintained glomerular diameter distributions comparable to their respective controls (Figure 3F–H).

### 2.6. MPR Protects Against Renal Fibrosis Following IR + Nx

Renal fibrosis was evaluated by morphometric analysis of sections stained with Sirius Red. As expected, and consistent with the renal dysfunction, proteinuria, and glomerular hypertrophy observed in the CC + IR group, this group exhibited extensive fibrosis, as shown in Figure 4B, compared with all control groups (Figure 4A,C,E,G), and with the CR + IR, RC + IR, and RR + IR groups (Figure 4D,F,H). Figure 4I shows the percentage change in fibrosis in the CC + IR, CR + IR, RC + IR, and RR + IR groups relative to their respective controls (CC, RC, CR, and RR), clearly indicating that the CC + IR group exhibited tubule-interstitial fibrosis.

### 2.7. MPR Attenuates Tubular Injury and Accelerates Functional Recovery Seven Days After AKI

Our findings indicate that the offspring of mothers subjected to protein restriction during gestation and/or lactation exhibited resilience in the transition from AKI to CKD. To explore the potential mechanisms underlying this long-term renoprotection, we evaluated the early response at 7 days post-IR in the different MPR groups. BW was comparable across all the studied groups (Figure 5A). First, to assess the severity of AKI, renal dysfunction, proteinuria, and markers of tubular injury were evaluated 24 h after IR +Nx. All rats subjected to IR + Nx showed a profound reduction in CrCl, with no differences among the dietary groups (Figure 5B). Proteinuria was increased at 24 h, but only the CC + IR and RC + IR groups reached statistical significance (Figure 5C). Tubular injury was further assessed through urinary excretion of renal biomarkers Kidney Injury Molecule-1 (KIM1) and Heat Shock Protein 72 (HSP72). Urinary KIM1 excretion was significantly higher in the CC + IR, CR + IR, RC + IR, and RR + IR groups compared to their respective control group and the other control groups. However, the highest KIM1 excretion was observed in the RC + IR group (Figure 5D). Consistently, this group also showed the greatest HSP72 excretion compared with the other IR + Nx groups (Figure 5E). After seven days, renal function was evaluated in this set of animals. Despite the absence of significant differences in MAP among the groups (Figure 5F), all rats subjected to IR + Nx showed a significant increase in kidney weight (Figure 5G). Interestingly, reduced CrCl persisted only in the CC + IR group, which was significantly lower than that observed in the CR + IR group (Figure 5H). No significant hypoperfusion was detected among the groups seven days after IR + Nx (Figure 5I).

### 2.8. MPR Suppresses Oxidative and Endoplasmic Reticulum Stress During Post-Ischemic Repair

In the early days of post-ischemic repair, we investigated possible pathways involved in the nephroprotection observed in offspring from mothers subjected to protein restriction during gestation and/or lactation. First, two markers of endoplasmic reticulum (ER) stress were analyzed. CHOP (C/EBP homologous protein) expression tended to be lower in the offspring from MPR with or without IR + Nx; however, only the RR + IR group reached statistical significance (Figure 6A). In the case of BiP (Binding Immunoglobulin Protein), the lowest expression was found in the RR + IR group (Figure 6B). When 4HNE (4-Hydroxynonenal) expression was assessed, the RR + IR group, which showed the highest degree of renoprotection, displayed a significant reduction in this oxidative stress marker compared to the CC + IR group (Figure 6C). Renal inflammation was assessed by measuring the expression of IL6 (Interleukin-6) and TNF-α (Tumor Necrosis Factor-alpha). All groups subjected to IR + Nx exhibited a significant reduction in IL6 expression compared to their respective control group and the control groups from MPR (Figure 6D). TNF-α expression was similar across all groups (Figure 6E). Finally, macrophage polarization toward the M2 phenotype was significantly increased in the CC + IR, CR + IR, and RR + IR groups compared with their control groups and the other control groups (Figure 6F).

## 3. Discussion

The present findings indicate that maternal exposure to a protein-restricted diet during gestation and/or lactation conferred enhanced resilience against the transition from AKI to CKD in the offspring. The CR + IR, RC + IR, and particularly the RR + IR groups showed attenuated proteinuria, absence of glomerular hypertrophy, and minimal fibrosis compared with the CC + IR group. Our previous study showed that MPR can attenuate acute tubular injury following IR + Nx [16]; the present findings extend these observations by demonstrating that this adaptive phenotype persists and is associated with protection against long-term renal deterioration.

We have previously shown that AKI-to-CKD progression in rats is characterized by progressive proteinuria, glomerular hypertrophy, and renal fibrosis [24,25,26,29]. In the present study, GFR was preserved five months after IR + Nx, including in CC + IR animals, despite proteinuria and structural alterations. This may reflect compensatory hyperfiltration and increased RBF following nephron loss after nephrectomy [30]. Although these mechanisms can preserve GFR, sustained hyperfiltration may promote glomerular injury. Consistent with this interpretation, marked glomerular hypertrophy and progressive proteinuria were observed in CC + IR animals but were largely absent in protein-restricted offspring. These findings are consistent with previous evidence that MPR during lactation reduces albuminuria in adult offspring [31].

The marked difference in tubulointerstitial fibrosis suggests that MPR influences not only the initial response to injury but also the subsequent repair process. Persistent oxidative stress, ER stress, and inflammation are important drivers of maladaptive repair and fibrosis after AKI [32,33]. In the acute cohort, RR + IR offspring showed reduced 4-HNE together with lower urinary KIM-1 and HSP72, suggesting reduced oxidative and tubular stress following IR + Nx. Nutritional restriction has previously been associated with reduced ROS generation and enhanced cellular stress resistance [34,35]. Thus, MPR may establish a renal phenotype with greater capacity to maintain redox homeostasis during ischemic stress.

ER stress may represent a complementary mechanism. Ischemia–reperfusion disrupts protein homeostasis and activates the unfolded protein response (UPR), which is initially adaptive but can promote cell death and fibrosis when sustained [36,37,38,39]. In protein-restricted offspring, BiP expression was significantly reduced and CHOP showed a decreasing trend, suggesting attenuation of ER stress. Because oxidative and ER stress are closely interconnected, reduced ROS generation may limit ER dysfunction, while reduced ER stress may decrease tubular cell death and inflammatory signaling. Together, these effects could favor adaptive repair and limit the chronic remodeling that characterizes AKI-to-CKD progression.

Systemic metabolic adaptations may also contribute to the renal resilience observed in the present study. MPR can induce persistent changes in offspring glucose and insulin metabolism, leptin physiology, lipid metabolism, hypothalamic–pituitary–adrenal function, and blood pressure [31,40,41]. These systemic adaptations may influence renal responses indirectly through changes in energy metabolism, vascular tone, sodium handling, and inflammatory regulation. Thus, the protection observed after IR + Nx may reflect coordinated systemic and renal adaptations rather than exclusively local renal mechanisms. Because systemic metabolic and hormonal parameters were not comprehensively assessed, their relative contribution to the observed phenotype cannot be determined. Future studies integrating systemic metabolic, endocrine, hemodynamic, immune, and renal profiling will be important to define how these pathways interact to promote renal resilience.

The inflammatory response may further contribute to this phenotype. Increased M2 macrophage polarization was observed in both CC + IR and RR + IR groups; therefore, M2 polarization alone cannot explain the protection associated with MPR. However, in RR + IR animals, increased M2 polarization occurred together with reduced 4-HNE and IL-6, suggesting a less oxidative and proinflammatory environment that may favor tissue repair. Further studies using temporal characterization and functional manipulation of macrophage populations are needed to establish their causal contribution.

From a developmental programming perspective, these findings highlight an apparent paradox. Although MPR reduces nephron endowment and is generally considered a risk factor for CKD, it can also reprogram metabolic and stress-response pathways that enhance resistance to subsequent renal injury [31,40,41]. Thus, nephron endowment alone may not determine renal vulnerability; developmental programming may also establish durable mechanisms of stress adaptation and tissue resilience. The persistence of this phenotype five months after IR + Nx suggests that these adaptations may influence the balance between adaptive and maladaptive repair long after the original nutritional exposure. Although these findings cannot yet be directly extrapolated to humans, they highlight the potential importance of developmental nutritional history as a factor in renal risk assessment. Future studies should determine the metabolic, epigenetic, and immune mechanisms underlying this renal resilience and identify biomarkers that may predict or promote adaptive repair after AKI.

Several limitations should be acknowledged. Although the protective phenotype persisted for five months after renal IR + Nx, longer follow-up and studies in aged offspring are needed to determine whether this resilience is maintained during aging. In addition, the contribution of systemic metabolic, endocrine, and immune adaptations, as well as the causal mechanisms underlying the observed protection, remains to be established. A further limitation is that MAP and RBF were measured under pentobarbital anesthesia, which can alter blood pressure and renal hemodynamics; therefore, absolute values may not fully reflect the physiological state of conscious animals. However, the same anesthetic regimen, dose, and experimental conditions were used across all groups, allowing reliable comparison of relative hemodynamic responses. Based on our previous experience, alternative anesthetic regimens produced more pronounced alterations in renal hemodynamics; therefore, pentobarbital was selected as a standardized regimen. These considerations should be taken into account when interpreting the hemodynamic findings.

In summary, our findings provide evidence that MPR programs a persistent renal phenotype that enhances the kidney’s resilience to acute injury and limits the progression from AKI to CKD. Notably, protein restriction during gestation and/or lactation did not alter baseline growth or the initial severity of ischemic kidney injury, yet was associated with substantial long-term protection against chronic renal deterioration. This phenotype was characterized by reduced oxidative and ER stress, preservation of glomerular architecture, attenuation of proteinuria, and reduced tubulointerstitial fibrosis. The significance of these findings extends beyond the established effects of MPR on nephron endowment and adult renal susceptibility, demonstrating that developmental nutritional history can also influence how the mature kidney responds to a subsequent acute insult and determines its long-term trajectory toward CKD. Together, these findings suggest that early-life nutritional programming can shape the kidney’s capacity for repair, favoring adaptive responses that promote recovery and restrain maladaptive progression toward CKD. Thus, developmental nutritional cues may not simply determine renal vulnerability through nephron endowment but may also establish durable mechanisms of stress adaptation and tissue resilience. Further studies are needed to define the molecular and epigenetic mechanisms underlying this programmed renal resilience and to determine whether these adaptations can be harnessed to prevent or attenuate AKI-to-CKD progression.

## 4. Materials and Methods

All experiments involving animals were conducted in accordance with the Nacional Institute for Health (NIH) Guide for the Care and Use of Laboratory Animals and with the Mexican Federal Regulation for animal reproduction, care, and experimentation (NOM-062-ZOO-2001). The study was carried out with the approval of the Animal Care Committee of our Institution (NMM-1720-15/18-1).

### 4.1. Subject Inclusion

For breeding, 50 female Wistar rats (220 ± 20 g) obtained from our institutional animal facility were used. Animals were housed under controlled environmental conditions (21–22 °C; 12:12 h light–dark cycle) and maintained on a standard laboratory diet (Purina Chow 5001) before mating. Females were housed in groups of three with one male, and gestational day 0 was defined by the presence of sperm in the vaginal smear. Pregnant rats were then assigned to one of two dietary groups: a control diet containing 20% casein (C) or an isocaloric protein-restricted diet containing 10% casein (R) as previously described [16] and illustrated in Figure 7.

During lactation and after the birth of the pups, dams were subjected to the experimental interventions described below and summarized in Figure 7:

***The control group (C)*** was subdivided into two groups: the CC group, which remained on the control diet throughout the study, and the CR group, which received a standard diet during pregnancy and was switched to a protein-restricted diet during lactation.
***The protein-restricted group (R)*** exposed to a low protein intake during pregnancy was also subdivided into two groups: the RR group, which continued on the protein-restricted diet during lactation, and the RC group, which was switched to a control diet during lactation.

Maternal BW was monitored throughout gestation and lactation (Table 1). At birth, litter size was standardized to 10 pups per dam from an initial range of 10–14 pups to ensure experimental consistency and equal sex distribution.

### 4.2. Weight and Morphometric Measurements of Pups

Between 12 and 24 h after birth, pups were weighed and morphometric measurements were obtained, including length (snout to tail tip), abdominal width (mid-abdominal transverse diameter), cranial width (interarticular distance), and anogenital distance for sex determination, using a Vernier caliper (Table 2). BW was subsequently monitored on postnatal days 7, 14, and 21 (Table 3).

### 4.3. Experimental Model

After weaning at postnatal day 21, offspring were separated by sex, and only males were included in the study. Male offspring were maintained on a standard rodent diet and assigned to experimental groups according to maternal dietary exposure (CC, CR, RC, and RR). Only male rats were studied because our previous work demonstrated that females are protected against the transition of AKI to CKD [19]. The sample size was determined a priori based on the variance estimates from a previous study from our laboratory [42] by assuming a 2.3-fold increase in the renal fibrosis % after 5 months post-ischemia compared to control group, with a two-sided α of 0.05 and 95% statistical power; the calculation yielded a minimum sample size of 7 animals after IR.

When the animals reached a BW of 280–330 g, they were randomly allocated within each maternal dietary group to one of two surgical conditions. One subgroup underwent left renal IR combined with right nephrectomy (Nx), generating the CC + IR, CR + IR, RC + IR, and RR + IR groups. The remaining animals underwent sham surgery with right Nx but without renal IR, generating the corresponding CC, CR, RC, and RR as control groups.

Each experimental group included two or three paternal half-siblings (i.e., offspring sharing the same father but not the same mother). This breeding design was used because institutional regulations and the principles of the 3Rs precluded the use of additional animals solely to ensure representation from multiple litters within each experimental group. The number of pups studied in each group is as follows: CC (n = 14), CR (n = 16), RC (n = 18), and RR (n = 19), CC + IR (n = 14), CR + IR (n = 14), RC + IR (n = 19), and RR + IR (n = 19). These animals were divided into two independent cohorts: one studied at 7 days and the other 5 months after surgery. For the short-term study, the sample sizes were CC (n = 4), CC + IR (n = 7), CR (n = 8), CR + IR (n = 5), RC (n = 9), RC + IR (n = 9), RR (n = 9), and RR + IR (n = 9). For the long-term study, the corresponding sample sizes were CC (n = 10), CC + IR (n = 7), CR (n = 9), CR + IR (n = 8), RC (n = 9), RC + IR (n = 10), RR (n = 10), and RR + IR (n = 10) (Figure 7).

Rats were anesthetized with sodium pentobarbital (30 mg/kg) and placed on a thermoregulated bed maintained at 37 °C. A midline laparotomy was performed to expose the retroperitoneal space. The left renal vascular bundle was carefully dissected, and RBF was interrupted by placing a vascular clip on the renal artery for 45 min. Simultaneously, the right renal vascular bundle was exposed, and the renal hilum was doubly ligated proximally and distally with 3-0 silk sutures. A cut was made between the ligatures, and the right kidney was removed without disturbing the adrenal gland. Complete hemostasis was confirmed. Renal ischemia was visually verified by the characteristic color change. Following the ischemic period, the abdominal muscle layer was sutured with 3-0 Vicryl, and the skin was closed using 3-0 silk. Once the animals recovered from anesthesia, they were placed in metabolic cages for a 24 h urine collection, followed by blood sampling from the retro-orbital plexus. Two experimental time points were evaluated: a short-term period at 7 days post-surgery and a long-term period at 5 months post-surgery to assess the progression from AKI to CKD (AKI-to-CKD transition). In the short-term group, an additional 24 h urine collection was performed on day 7. In the 5-month AKI-to-CKD group, BW was recorded, and urine samples were collected monthly. At five months post-surgery, the physical condition of the animals was evaluated by magnetic resonance imaging (MRI; Echo Medical Systems, Houston, TX, USA), and a final 24 h urine collection was obtained.

### 4.4. Physiological Hemodynamics

At seven days or five months post-surgery, rats were again re-anesthetized with sodium pentobarbital (30 mg/kg) and placed on a thermoregulatory table maintained at 37 °C. To measure MAP, both femoral arteries were catheterized and one of them was connected to a pressure transducer (model p23) coupled to a polygraph (Grass Instrument, Quincy, MA, USA). A laparotomy was then performed and RBF was recorded by placing an ultrasound flow probe with acoustic gel around the left renal artery connected to a flowmeter (Transonic Flow Probe, New York, NY, USA). Following physiological measurements, blood samples were collected from the catheterized femoral artery into heparinized tubes. Rats were then euthanized, and kidney tissue was harvested for subsequent analysis.

### 4.5. Assessment of Proteinuria, Plasma Creatinine, and CrCl

Proteinuria was assessed from 24 h urine samples collected at 24 h and seven days after IR + Nx or sham surgery in the short-term period cohort, while it was measured monthly in the long-term cohort. Protein concentration was determined using the turbidimetric method with trichloroacetic acid [43].

Before the euthanasia, blood samples were obtained and centrifuged at 10,000 rpm for 10 min to separate plasma. Plasma and urine creatinine concentrations were measured using a Quantichrom kit (DICT-500, BioAssay System, Hayward, CA, USA) according to the manufacturer’s instructions. CrCl was calculated using the following formula: CrCl = (U × V)/P, where U is urine creatinine concentration, V is urine flow rate, and P is plasma creatinine concentration.

### 4.6. Urinary Biomarkers of Kidney Damage

Proteins were separated by SDS-PAGE using an 8.5% acrylamide gel, loading 0.5 μL of urine for HSP72 and 1.5 μL for KIM1. Proteins were transferred to polyvinylidene fluoride (PVDF) membranes using a Trans-Blot Turbo Transfer System (Bio-Rad Laboratories, Hercules, CA, USA) for 25 min at 25 V. Membranes were blocked with a 5% milk for 60 min and incubated overnight at 4 °C with anti-HSP72 antibody (ENZO Life Science, Farmingdale, NY, USA; 1:5000) or anti-KIM-1 antibody (Biorbyt, Cambridge, UK; 1:1000). After washing, membranes were incubated with anti-mouse secondary antibody (Santa Cruz Biotechnology, Inc., Dallas, TX, USA; 1:5000) at room temperature for 90 min. Proteins were detected using a chemiluminescent kit (Merck Millipore, Burlington, MA, USA) and visualized on an iBright Imaging System (iBright FL1500, Thermo Fisher Scientific, Waltham, MA, USA). Band intensity was quantified by densitometry using iBright Analysis Software version 5.3.0 (Thermo Fisher Scientific, Waltham, MA, USA). 

### 4.7. Renal Expression of Markers of Inflammation and Endoplasmic Reticulum Stress

Total renal proteins were isolated from the renal cortex of each rat and homogenized in a lysis buffer (50 mM HEPES pH 7.4, 250 mM NaCl, 5 mM EDTA, 0.1% NP-40, and complete protease inhibitor (Roche Diagnostics GmbH, Mannheim, Germany)). Protein samples were resolved by 8.5% SDS-PAGE electrophoresis and electroblotted into PVDF membranes (Merck Millipore, Burlington, MA, USA). Membranes were then blocked with 5% blotting-grade non-fat dry milk. The membranes were then incubated in 5% blotting-grade non-fat dry milk with their respective antibodies overnight at 4 °C. Specific antibodies against CD206 (Abcam, Cambridge, UK, 1:5000 dilution), IL-6 (Santa Cruz Biotechnology, Inc., Dallas, TX, USA, 1:750 dilution), 4HNE (Abcam, Cambridge, UK, 1:500 dilution), CHOP (Cell Signaling Technology, Danvers, MA, USA, 1:5000 dilution), BiP (Cell Signaling Technology, Danvers, MA, USA, 1:2500 dilution), and TNFα (Cell Signaling Technology, Danvers, MA, USA, 1:1000 dilution) were used. After incubation with the primary antibody, the membranes were washed and incubated with their respective secondary antibodies, anti-rabbit (SIGMA, Sigma-Aldrich, St. Louis, MO, USA, 1:5000 dilution) or anti-mouse (Jackson ImmunoResearch Laboratories, Inc., West Grove, PA, USA, 1:5000 dilution), at room temperature for 90 min. As a loading control, the membranes were incubated overnight at 4 °C with actin antibody conjugated to Horseradish peroxidase (Abcam, Cambridge, UK, 1:1,500,000 dilution). Proteins were detected as indicated above. 

### 4.8. Histomorphometry and Histopathological Studies

After recording the physiological parameters, a segment of each left kidney was perfused through the femoral catheter with 0.9% NaCl and with 4% formalin. The kidney tissue was then embedded in paraffin, sectioned at 3 μm thickness, and stained with periodic acid Schiff (PAS) and Sirius Red. Microphotographs of ten randomly selected fields from the renal cortex (200× magnification) were captured using using a Nikon microscope equipped with a digital camera (Nikon Corporation, Tokyo, Japan). All slide analyses were performed in a blinded manner. Fibrosis, visualized as a red staining with Sirius Red, was quantified, and the % of fibrosis was calculated by dividing the fibrotic area by the total area in each slide. Fibrotic area and glomerular diameter were analyzed in the 5-month experiments using NIS-Elements AR software, version 4.5 (Nikon Corporation, Tokyo, Japan). 

### 4.9. Statistical Analysis

Data are presented as mean ± SD and were analyzed using two-way ANOVA. Group differences were assessed using the two-stage step-up post hoc test (Benjamini, Krieger, and Yekutieli) for multiple comparisons. The distribution of glomerular diameters was evaluated by contingency analysis. The percentage of fibrosis, relative to each respective control group, was analyzed by one-way ANOVA followed by Bonferroni’s multiple-comparisons test. Differences were considered statistically significant at *p* < 0.05.

## Figures and Tables

**Figure 1:**
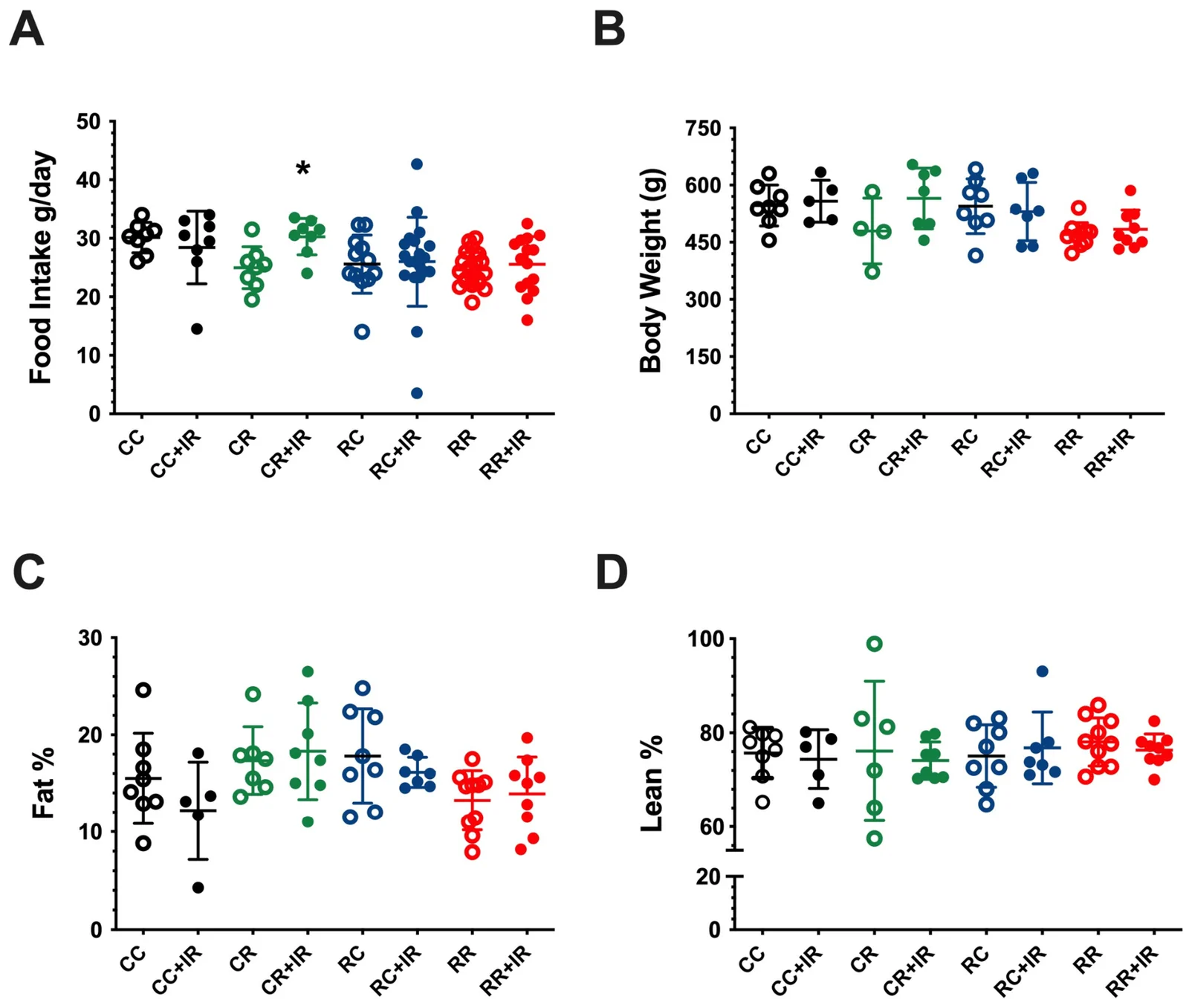
**Food Intake, BW, and Body Composition in all studied groups**. (**A**) Food intake, (**B**) BW, (**C**) fat percentage, and (**D**) lean mass percentage in offspring from mothers fed control (CC), control during gestation and restricted during lactation (CR), restricted during gestation and control during lactation (RC), or restricted during both periods (RR) diets. White and black circles represent the CC group without and with IR + Nx, respectively; open and filled green circles represent the CR group without and with IR + Nx, respectively; open and filled blue circles represent the RC group without and with IR + Nx, respectively; and open and filled red circles represent the RR group without and with IR + Nx, respectively. Data are expressed as mean ± SD; * *p* < 0.05 vs. its respective control group.

**Figure 2:**
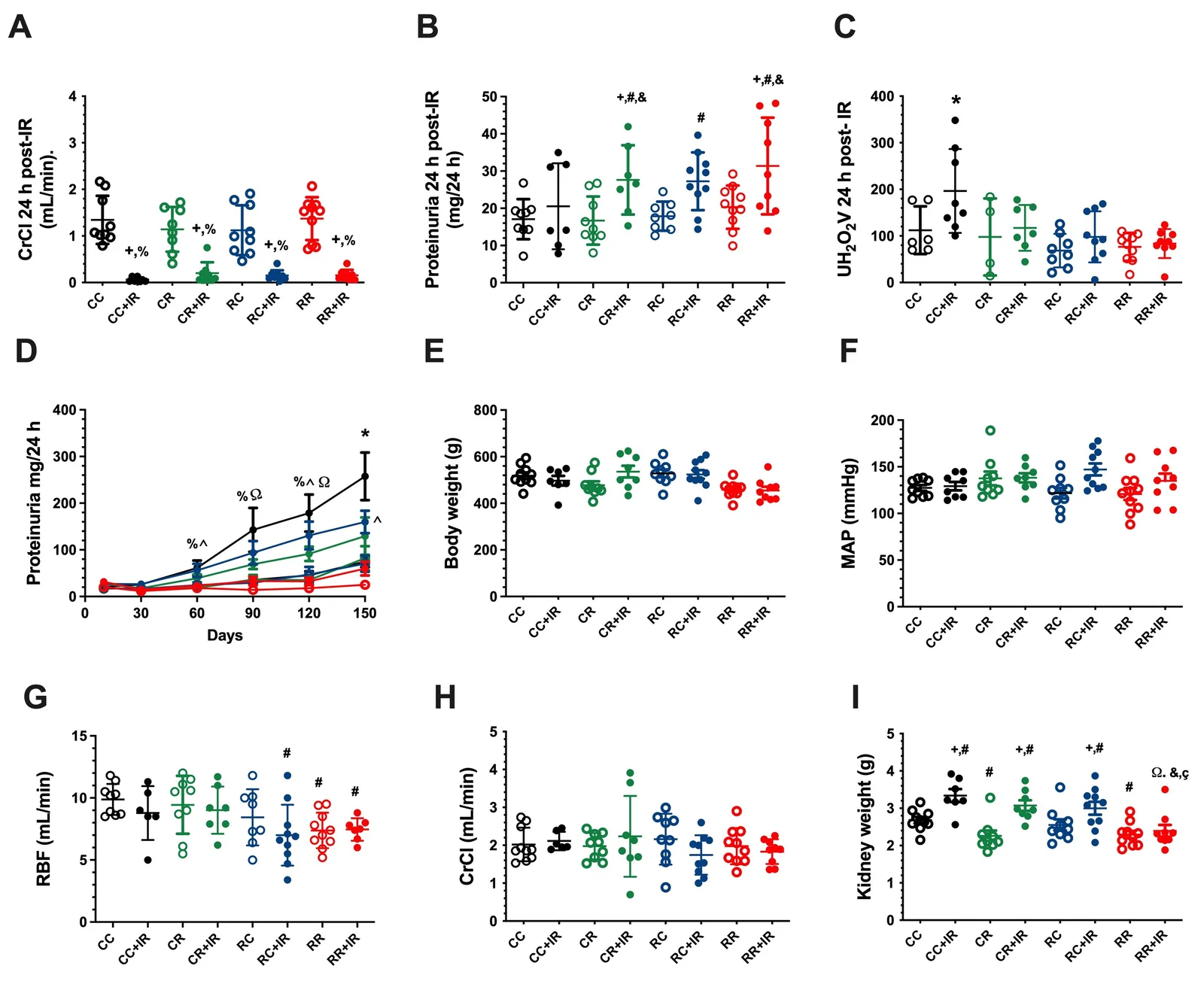
**Renal Function in the long-term cohort after AKI induction.** (**A**–**C**) CrCl, proteinuria, and urinary H_2_O_2_ excretion measured after 24 h of IR + Nx injury. (**D**–**I**) Long-term follow-up of the same animals over 5 months showing (**D**) proteinuria, (**E**) BW, (**F**) mean arterial pressure (MAP), (**G**) RBF, (**H**) CrCl, and (**I**) kidney weight. White and black circles represent offspring of the CC group without and with IR + Nx, respectively; open and filled green circles represent the CR group without and with IR + Nx, respectively; open and filled blue circles represent the RC group without and with IR + Nx, respectively; and open and filled red circles represent the RR group without and with IR + Nx, respectively. Data are expressed as mean ± SD; each group included at least seven rats. * *p* < 0.05 vs. all the groups (CC, CR, RC, RR), % *p* < 0.05 vs. all control groups, + *p* < 0.05 vs. its respective control group, # *p* < 0.05 vs. all other control groups, & *p* < 0.05 vs. the CC + IR group, ^ *p* < 0.05 vs. the RR + IR group, Ω *p* < 0.05 vs. the CR + IR group, and Ç *p* < 0.05 vs. the RC + IR group.

**Figure 3:**
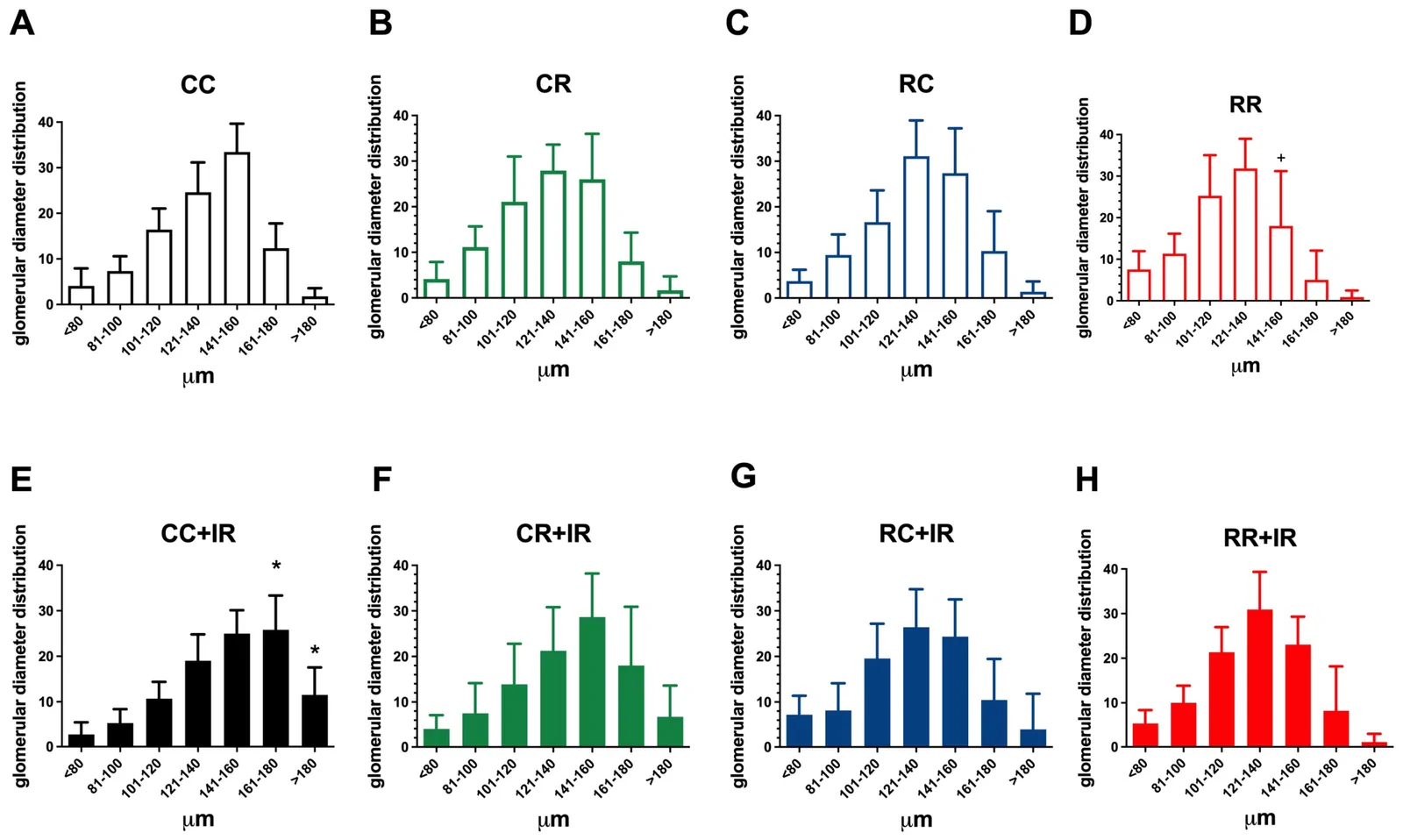
**Glomerular hypertrophy was observed in the CC + IR group, but not in the offspring of maternal protein-restricted groups.** (**A**–**D**) Glomerular diameter distribution in control groups: the CC, CR, RC, and RR, respectively. (**E**–**H**) Glomerular diameter distribution in the corresponding IR + Nx groups: CC + IR, CR + IR, RC + IR, and RR + IR, respectively. * *p* < 0.05 vs. its respective control group and + *p* < 0.05 vs. the CC group.

**Figure 4:**
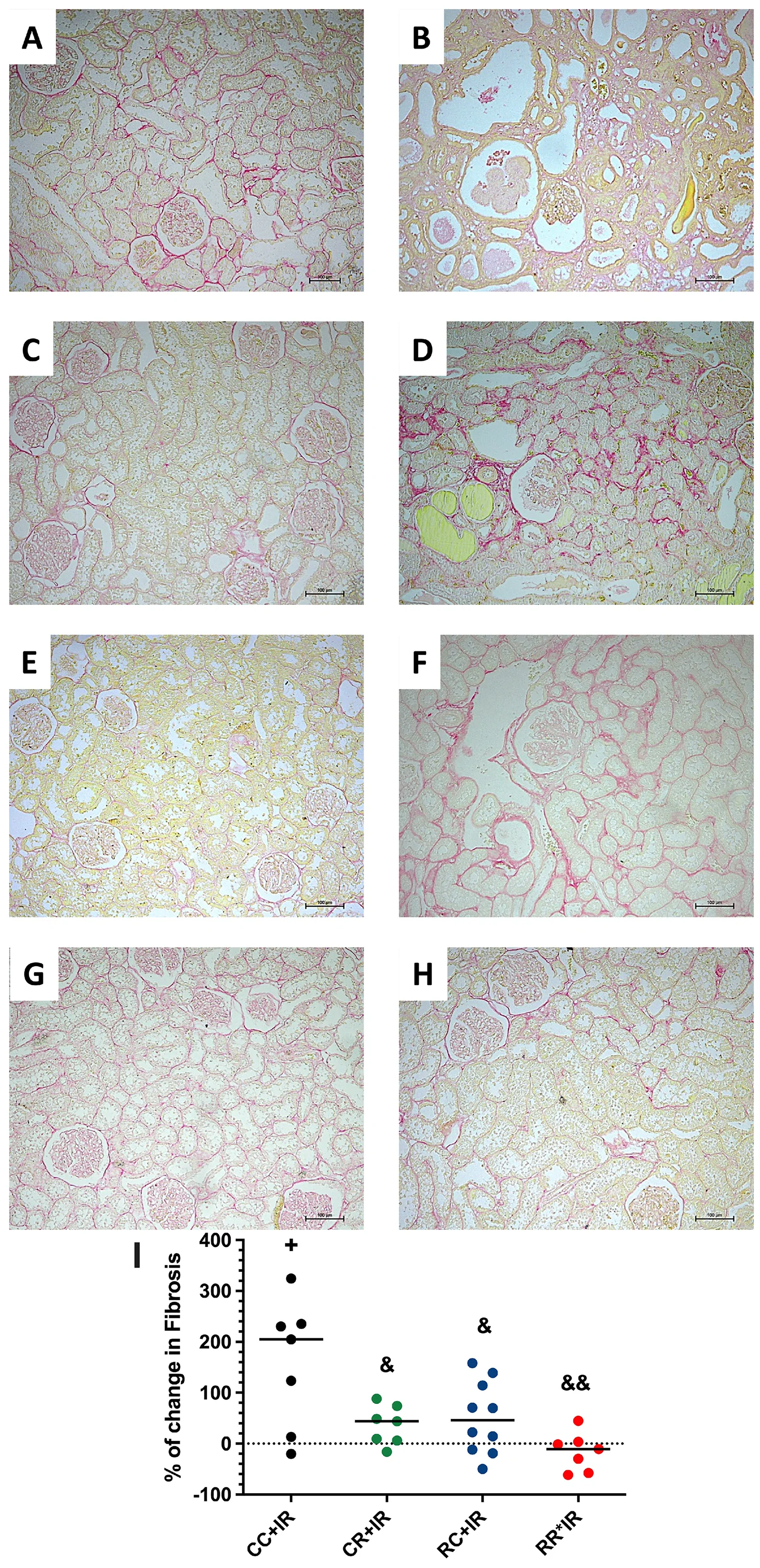
**Renal fibrosis in offspring following ischemia-reperfusion (IR) injury.** Significant fibrosis was observed in the CC + IR group, whereas offspring of maternal protein-restricted groups exhibited minimal fibrosis. Representative microphotographs of kidney sections stained with Picrosirius Red are shown for (**A**) Control (CC), (**B**) CC + IR, (**C**) CR, (**D**) CR + IR, (**E**) RC, (**F**) RC + IR, (**G**) RR, (**H**) RR + IR. Panel (**I**) shows the percentage change in renal fibrosis compared with the respective control groups. + *p* < 0.05 vs. its respective control group, & *p* < 0.05 vs. the CC + IR, && *p* < 0.01 vs. the CC + IR.

**Figure 5:**
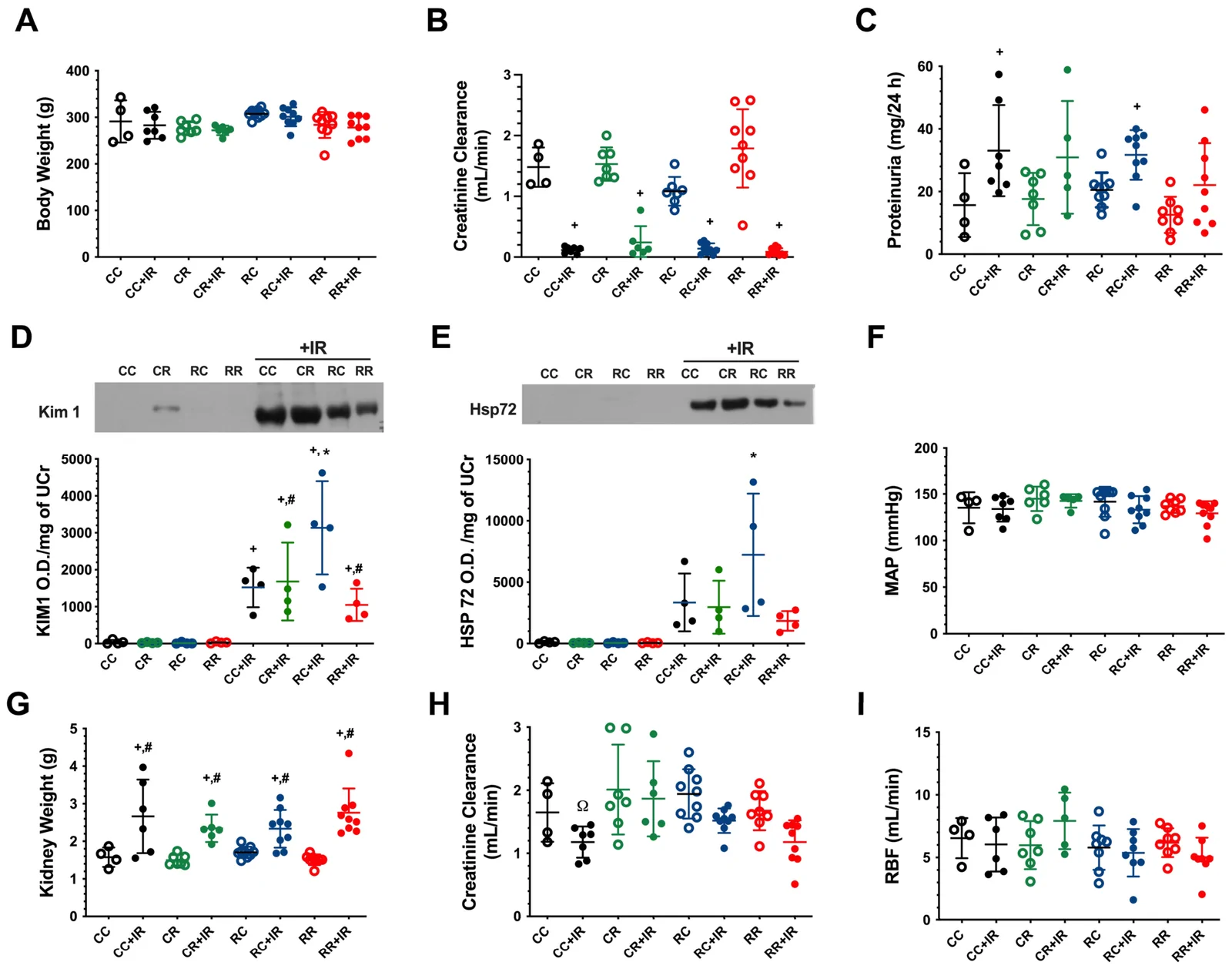
**Renal physiological parameters in the short term following IR + Nx injury**. (**A**) BW, (**B**) CrCl evaluated 24 h after IR + Nx, (**C**) Proteinuria assessed 24 h after IR + Nx, (**D**,**E**) Urinary excretion of KIM and HSP72, respectively, (**F**) MAP measured seven days after IR + Nx, (**G**) Kidney weight seven days after IR + Nx, (**H**) CrCl, and (**I**) RBF recorded seven days after IR + Nx. White and black circles represent the CC group without and with IR + Nx, respectively; open and filled green circles represent the CR group without and with IR + Nx, respectively; open and filled blue circles represent the RC group without and with IR + Nx, respectively; and open and filled red circles represent the RR group without and with IR + Nx, respectively. Data are expressed as mean ± SD. * *p* < 0.05 vs. all the groups, + *p* < 0.05 vs. its respective control group, # *p* < 0.05 vs. other control groups, and Ω *p* < 0.05 vs. the CR + IR group.

**Figure 6:**
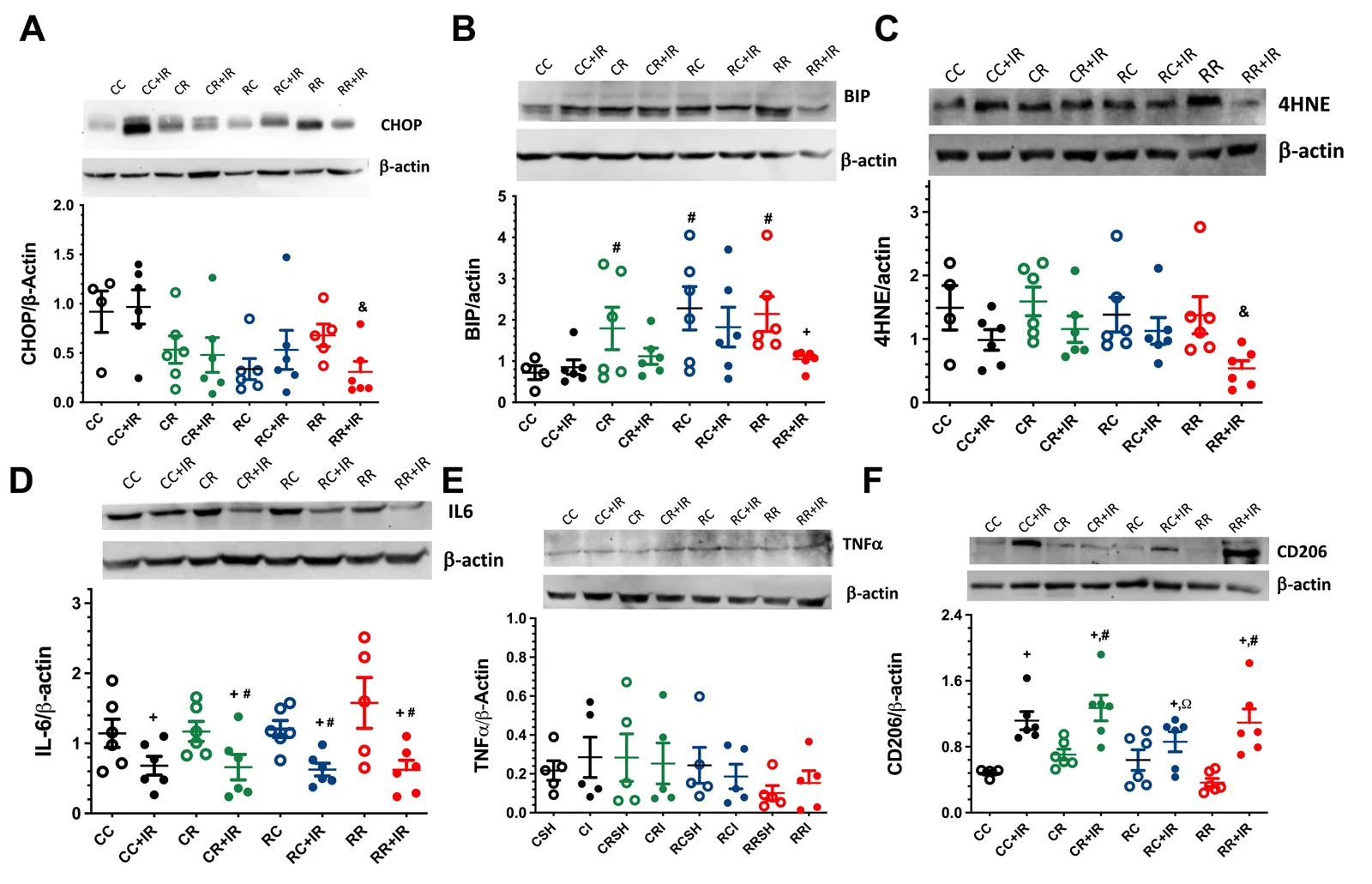
**Renal expression of markers of endoplasmic reticulum stress, oxidative stress, and inflammation in the short-term following IR + Nx injury.** (**A**) CHOP, (**B**) BiP, (**C**) 4-HNE, (**D**) IL-6, (**E**) TNFα, and (**F**) CD206. White and black circles represent the CC group without and with IR + Nx, respectively; open and filled green circles represent the CR group without and with IR + Nx, respectively; open and filled blue circles represent the RC group without and with IR + Nx, respectively; and open and filled red circles represent the RR group without and with IR + Nx, respectively. Data are expressed as mean ± SD. + *p* < 0.05 vs. its respective control group, # *p* < 0.05 vs. compared with all other control groups, Ω *p* < 0.05 vs. the CR + IR group, and & *p* < 0.05 vs. the CC + IR group.

**Figure 7:**
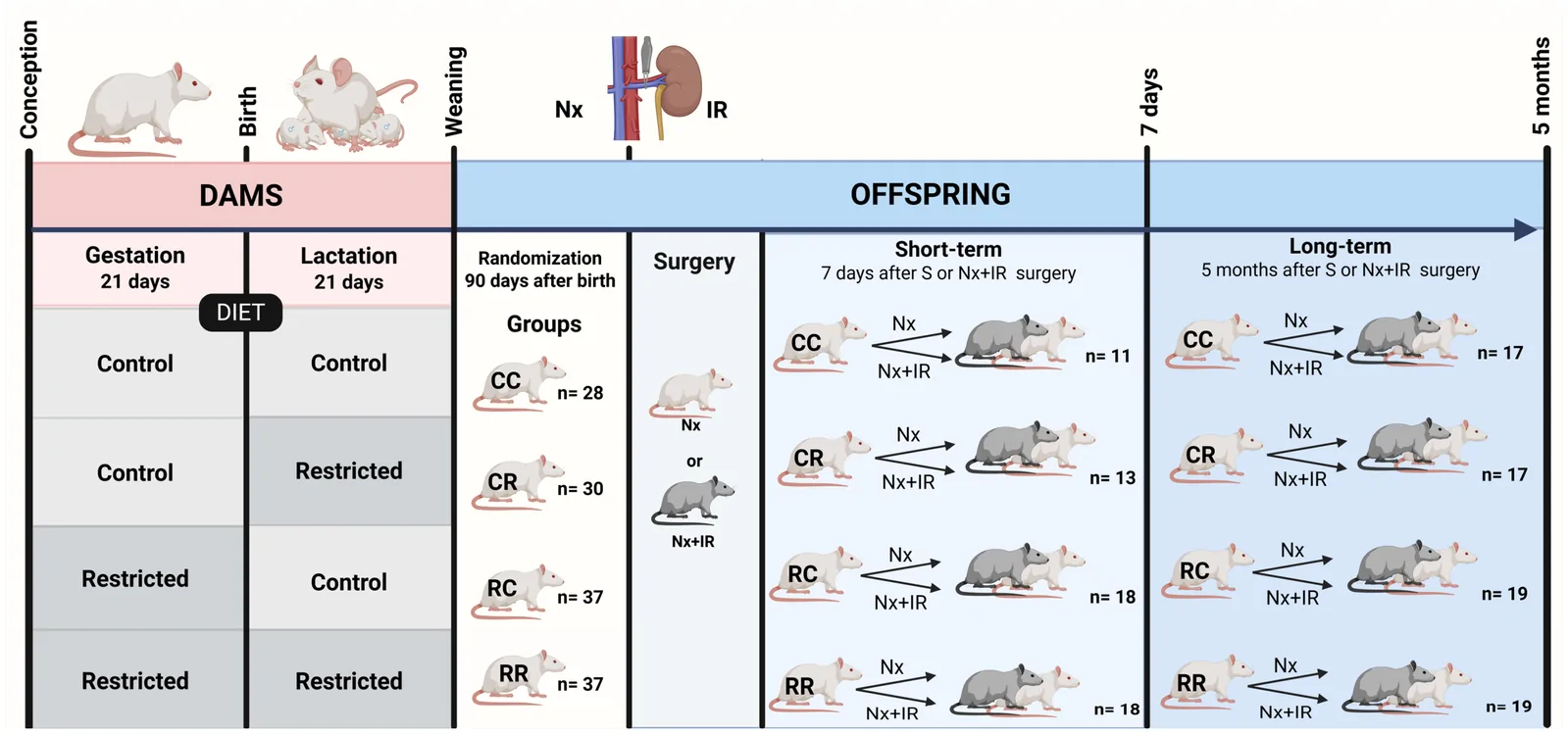
**Experimental design.** Female rats were housed with males, and the presence of spermatozoa in vaginal smears was designated as day 0 of gestation. Pregnant rats were randomly assigned to one of two dietary groups: a control diet (C) or a protein-restricted diet (R). After delivery and throughout the lactation period, control mothers were divided into two subgroups: one continued on a control diet (group CC), whereas the other received a protein-restricted diet (group CR). Similarly, mothers fed the protein-restricted diet during gestation were subdivided into two groups: one switched to the control diet (group RC), and the other remained on the protein-restricted diet (group RR). The offspring of these maternal groups formed the CC, CR, RC, and RR groups. At 90 days of age, all offspring underwent sham or the ischemia/reperfusion + nephrectomy (IR + Nx) procedure. Physiological parameters, blood, and tissue samples were collected and analyzed in two cohorts: short-term (7 days post IR + Nx) and long-term (5 months post IR + Nx).

**Table 1:** MaternalBW monitoring during gestation and lactation in the control group (CC), protein-restricted diet during lactation (CR), gestation (RC), or both (RR).

**Maternal BW Trajectory During Gestation**					
**Group**	**1st Day**	**5th Day**	**10th Day**	**15th Day**	**20th Day**
CC	252.0 ± 35.8	269.0 ± 41.2 *	296.0 ± 33.6 *	322.0 ± 46.8 *	375.0 ± 39.1 *
CR	250.0 ± 23.7	284.0 ± 20.1	310.0 ± 20.1 *	345.0 ± 24.5 *	394.0 ± 24.3 *
RC	263.0 ± 29.7	286.0 ± 30.0	300.0 ± 32.7	328.0 ± 41.7 *	380.0 ± 29.0 *
RR	286.0 ± 30.0	295.0 ± 16.1	294.0 ± 22.8	319.0 ± 41.7 *	381.0 ± 33.2 *
**Maternal BW Trajectory During Lactation**					
**Group**	**1st Day**	**5th Day**	**10th Day**	**15th Day**	**20th Day**
CC	282.0 ± 33.7	280.0 ± 34.1	304.0 ± 29.9	293.0 ± 31.6	282.0 ± 32.3
CR	305.0 ± 23.0	299.0 ± 23.4	297.0 ± 27.2	290.0 ± 26.9	291.0 ± 26.8
RC	302.0 ± 34.6	309.0 ± 31.9	327.0 ± 25.7	316.0 ± 26.2	317.0 ± 27.4
RR	292.0 ± 25.4	295.0 ± 16.1	289.0 ± 16.3	293.0 ± 5.2	291.0 ± 11.3

Control group (CC), protein-restricted diet during lactation (CR), gestation (RC), or both (RR) groups. Data are presented as mean ± SD. * *p* < 0.05 indicates a statistically significant difference compared with the respective value on day 1.

**Table 2:** Postnatal weight and morphometric parameters of rat pups from the studied experimental groups.

Group	Birth Weight (g)	Body Length (cm)	Body Width (cm)	Cranial Width (cm)
CC	6.5 ± 0.58	6.8 ± 0.46	1.49 ± 0.16	1.21 ± 0.09
CR	6.3 ± 0.76	7.0 ± 0.59	1.46 ± 0.12	1.19 ± 0.10
RC	6.9 ± 0.82	7.0 ± 0.59	1.54 ± 0.15	1.23 ± 0.08
RR	6.5 ± 0.82	6.8 ± 0.41	1.51 ± 0.02	1.23 ± 0.07

Control group (CC), protein-restricted diet during lactation (CR), gestation (RC), or both (RR) groups. Data are shown as mean ± SD.

**Table 3:** BW of Rat Pups During the First 30 Days of Postnatal Life.

Group	Day 7	Day 14	Day 21	Day 30
CC	16.7 ± 2.1	33.5 ± 5.0	55.4 ± 8.3	109.8 ± 12.8
CR	14.7 ± 2.8 *	27.4 ± 6.7 *	43.7 ± 8.6 *	98.4 ± 12.9 *
RC	18.05 ± 2.0 *	35.17 ± 4.0	57.9 ± 5.5	111.6 ± 13.8
RR	14.8 ± 1.8 *	26.0 ± 3.4 *	37.9 ± 6.9 *	85.5 ± 17.0 *

Control (CC), protein-restricted diet during lactation (CR), gestation (RC), or both (RR) groups. Data are shown as mean ± SD. * indicates *p* < 0.05 vs. the CC group.

## Data Availability

The original contributions presented in this study are included in the article and Supplementary Materials. Further inquiries can be directed to the corresponding author.

## Supplementary Materials

The following supporting information can be downloaded at https://www.mdpi.com/article/10.3390/ijms27208974/s1.

## Abbreviations

The following abbreviations are used in this manuscript:

4-HNE	4-Hydroxynonenal
AKI	Acute Kidney Injury
α-SMA	Alpha-Smooth Muscle Actin
BiP	Binding Immunoglobulin Protein
BW	Body Weight
C	Control
CC	Control group during both gestation and lactation
CD206	Cluster of Differentiation 206
CHOP	C/EBP Homologous Protein
CKD	Chronic Kidney Disease
CR	Control diet during gestation and protein-Restricted diet during lactation
CrCl	Creatinine Clearance
ER	Endoplasmic Reticulum
GFR	Glomerular Filtration Rate
H_2_O_2_	Hydrogen Peroxide
HIF1-α	Hypoxia-Inducible Factor 1-alpha
HSP72	Heat Shock Protein 72
IL-6	Interleukin-6
IR	Ischemia-Reperfusion
IR + Nx	Ischemia-Reperfusion + nephrectomy
KIM1	Kidney Injury Molecule-1
MAP	Mean Arterial Pressure
MPR	Maternal Protein Restriction
MRI	Magnetic Resonance Imaging
NIH	National Institutes of Health
Nx	Nephrectomy
PAS	Periodic Acid-Schiff
PVDF	Polyvinylidene Fluoride
R	Protein-Restricted diet
RBF	Renal Blood Flow
RC	Protein-Restricted diet during gestation and Control diet during lactation
ROS	Reactive Oxygen Species
RR	Protein-Restricted diet during both gestation and lactation
S	Sham (surgery)
SD	Standard Deviation
SDS-PAGE	Sodium Dodecyl Sulfate-Polyacrylamide Gel Electrophoresis
TGF-β	Transforming Growth Factor-beta
TNF-α	Tumor Necrosis Factor-alpha
UH_2_O_2_V	Urinary Excretion Rate of Hydrogen Peroxide
UPR	Unfolded Protein Response
