# Supplementary material for: Nutritional Programming via Maternal Protein Restriction Mitigates Long-Term Consequences of Acute Kidney Injury
Source: Int J Mol Sci. 2026 Oct 10;27(20):8974. doi: 10.3390/ijms27208974 (PMC13704265; doi:10.3390/ijms27208974)

Figure S1. Growth and morphometric characteristics of offspring at birth.

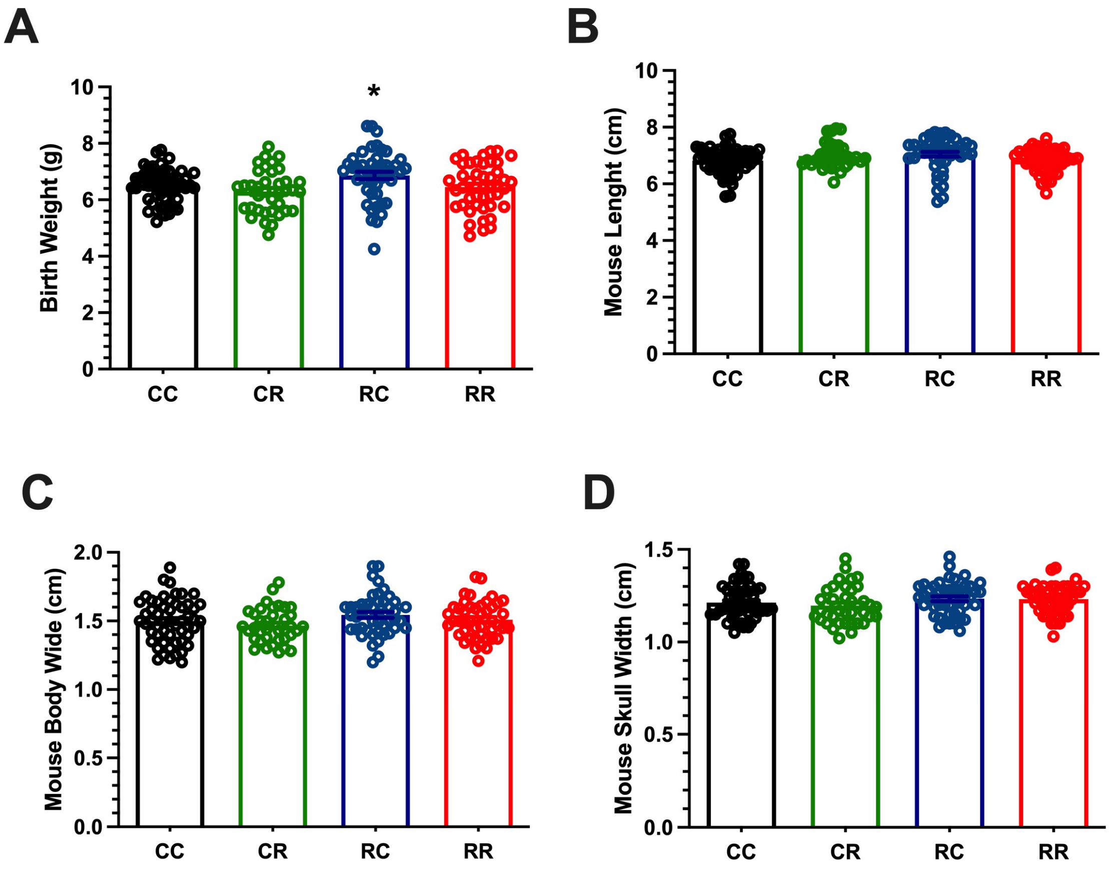

Supplement: Supplementary file 1 [file ijms-27-08974-s001.zip › ijms-4547533-supplementary.pdf]
